# 

**Published:** 1871-10

**Authors:** 


### CONTENTS.

COMMUNICATIONS.

Are the Mineral Acids formed in the mouth? ... 413
Dental Jottings ... 427
Operative Dentistry, or Filling Teeth ... 437

PROCEEDINGS OF SOCIETIES.

Proceedings of the Wisconsin State Dental Society ... 444

EDITORIAL.

The Brooklyn Dental Society ... 452
Mad River Dental Society ... 452
Change and Removal ... 453
The Chicago Fire ... 454
Dental Education.—Twenty-sixth Annual Session Ohio College of Dental Surgery ... 454

